# Lesions of abdominal connectives reveal a conserved organization of the calling song central pattern generator (CPG) network in different cricket species

**DOI:** 10.1007/s00359-021-01495-1

**Published:** 2021-06-07

**Authors:** Chu-Cheng Lin, Berthold Hedwig

**Affiliations:** Department of Zoology, University of Cambridge, Downing Street, Cambridge, CB2 3EJ UK

**Keywords:** Abdominal ganglia, Lesions, Central pattern generation, Calling song, Cricket

## Abstract

Although crickets move their front wings for sound production, the abdominal ganglia house the network of the singing central pattern generator. We compared the effects of specific lesions to the connectives of the abdominal ganglion chain on calling song activity in four different species of crickets, generating very different pulse patterns in their calling songs. In all species, singing activity was abolished after the connectives between the metathoracic ganglion complex and the first abdominal ganglion A3 were severed. The song structure was lost and males generated only single sound pulses when connectives between A3 and A4 were cut. Severing connectives between A4 and A5 had no effect in the trilling species, it led to an extension of chirps in a chirping species and to a loss of the phrase structure in two *Teleogryllus* species. Cutting the connectives between A5 and A6 caused no or minor changes in singing activity. In spite of the species-specific pulse patterns of calling songs, our data indicate a conserved organisation of the calling song motor pattern generating network. The generation of pulses is controlled by ganglia A3 and A4 while A4 and A5 provide the timing information for the chirp and/or phrase structure of the song.

## Introduction

Rhythmic movements are characteristic for behaviours like swimming, crawling, flying, walking, ventilation or sound production in a variety of invertebrates and vertebrates. The underlying motor patterns are generated by neural circuits called central pattern generators (CPGs) that once activated, produce the neural activity driving the motor output of the central nervous system (CNS) (Delcomyn 1980; Katz 2016). The organization of the pattern generators is in the focus of neuroscience, aiming to unravel the general principles of their function (Selverston 1980, 2010; Marder and Bucher 2001; Bucher et al. 2015). Central questions in these studies are: Where are the CPG networks located within the CNS and how are they organized at a systems and cellular level? Unravelling the network organisation may be straight forward, when the motor pattern is generated in a confined ganglion like for foregut movements (Ayali 2004) but it becomes more challenging, when the pattern is based on coordinated activity of at least two ganglia like in locust flight (Wilson 1961; Robertson and Pearson 1984), or involves all thoracic ganglia in generating the leg walking motor pattern (Bidaye et al. 2018; Mantziaris et al. 2017; Knebel et al. 2019), or the motor pattern is coordinated and distributed over several segments of the animal’s body like in ventilation (Lewis et al. 1973; Ramirez and Pearson 1989) and in the locomotor activity of larval Drosophila (Pulver et al. 2015).

Besides electrophysiological studies at a cellular level, lesion experiments have substantially contributed to our understanding of behaviour and the organization of the underlying neural networks. A hemiganglionic organisation of the flight pattern generator in thoracic ganglia was demonstrated for the locust flight system by means of severing pairs of connectives and hemisection of thoracic ganglia (Wolf et al. 1988; Ronacher et al. 1988). In the study of grasshopper sound production lesions narrowed down the ganglia housing the singing-CPG to the 2^nd^ and 3^rd^ thoracic ganglion (Huber 1960; Hedwig 1986), while a hemi-ganglionic organization of the singing-CPG in the 3^rd^ thoracic ganglion was revealed by longitudinal lesions (Ronacher 1989, 1991; Fries and Elsner 1996; Heinrich and Elsner 1997).

Unravelling the organization of the cricket singing-CPG turned out to be more cumbersome. Male crickets produce a calling song by opening and closing the forewings in a rhythmic and coordinated manner with specialized structures on the forewings sweeping against each other (Bennet-Clark 1989; Koch et al. 1988; Jonsson et al. 2021). Each closing movement of the elevated wings generates a sound pulse, and several pulses may be grouped in trills, chirps or phrases, with at least 2–3 rhythms involved in shaping complex song patterns (Otte 1992). For some time it was proposed that the CPG for cricket calling song is located in the mesothoracic ganglion, where the motor neurons innervating the wing muscles are housed (Huber 1960, 1963; Elepfandt 1980). However, accumulating experimental evidence questioned this conclusion and pointed to the importance of the abdominal nervous system in generating the calling song motor activity. The mesothoracic ganglion in *G. bimaculatus* can be split along its midline, without altering the calling song pattern (Hennig and Otto 1996). Male *G. campestris* sang after the cervical connectives were cut, but failed to sing if the cervical connectives and the connectives posterior to the thoracic ganglia were severed (Kutsch and Otto 1972). In *G. firmus*, recording of songs under differential heating conditions of the insects body suggested the involvement of abdominal ganglia in the control of singing motor activity (Pires and Hoy 1992). These results triggered new experiments analyzing the organization of the singing network with acute lesions to the abdominal ganglion chain (Schöneich and Hedwig 2011) and combining lesions with long-term recordings of cricket calling songs (Jacob and Hedwig 2016). In *G. bimaculatus*, lesions to the abdominal nerve cord implicated a different role of each abdominal ganglion for the generation of pulses and chirps, which define the calling song structure (Jacob and Hedwig 2016). These findings showcase the importance of the abdominal ganglia in cricket song pattern generation and have been supported by intracellular studies of singing interneurons recorded and identified in the abdominal ganglia (Schöneich and Hedwig 2011, 2012; Jacob and Hedwig 2019, 2020).

Here we evaluate the possible contribution of abdominal ganglia to the calling song structure and temporal organization of the pulse pattern in different species. We applied systematic lesions to the abdominal nerve cord of four cricket species with different species-specific calling song types: *Gryllus rubens* as trill-producing species, *G. assimilis* as chirp-producing species, and *Teleogryllus oceanicus* and *T. commodus* as phrase-producing species. The change of the calling song after each lesion was compared to the normal singing behaviour and provides evidence on the organization and evolution of the cricket song pattern generating neural system.

## Materials and methods

### Experimental animals

Colonies of four cricket species (*G. rubens, G. assimilis, T. oceanicus and T. commodus*) were kept in the Department of Zoology, University of Cambridge. Crickets lived in boxes (52.5 × 36.5 × 28 cm), last instar male nymphs were separated and kept solitary in boxes (17.5 × 11.5 × 13 cm) to monitor their development and age until sexual maturity. Fish food, muesli, potatoes and water were provided on a daily basis. Crickets were raised at 26–28 °C with a 12 h–12 h light:dark cycle. Adults seven to fourteen- days old after imaginal eclosion were selected for sound recording and lesion experiments. All animal treatments and experiments complied with the principles of Laboratory Animal Care (ASAB Ethics Committee and ABS Animal Care Committee 2021).

### Selective lesions of the abdominal central nervous system (CNS)

**Fig. 1:**
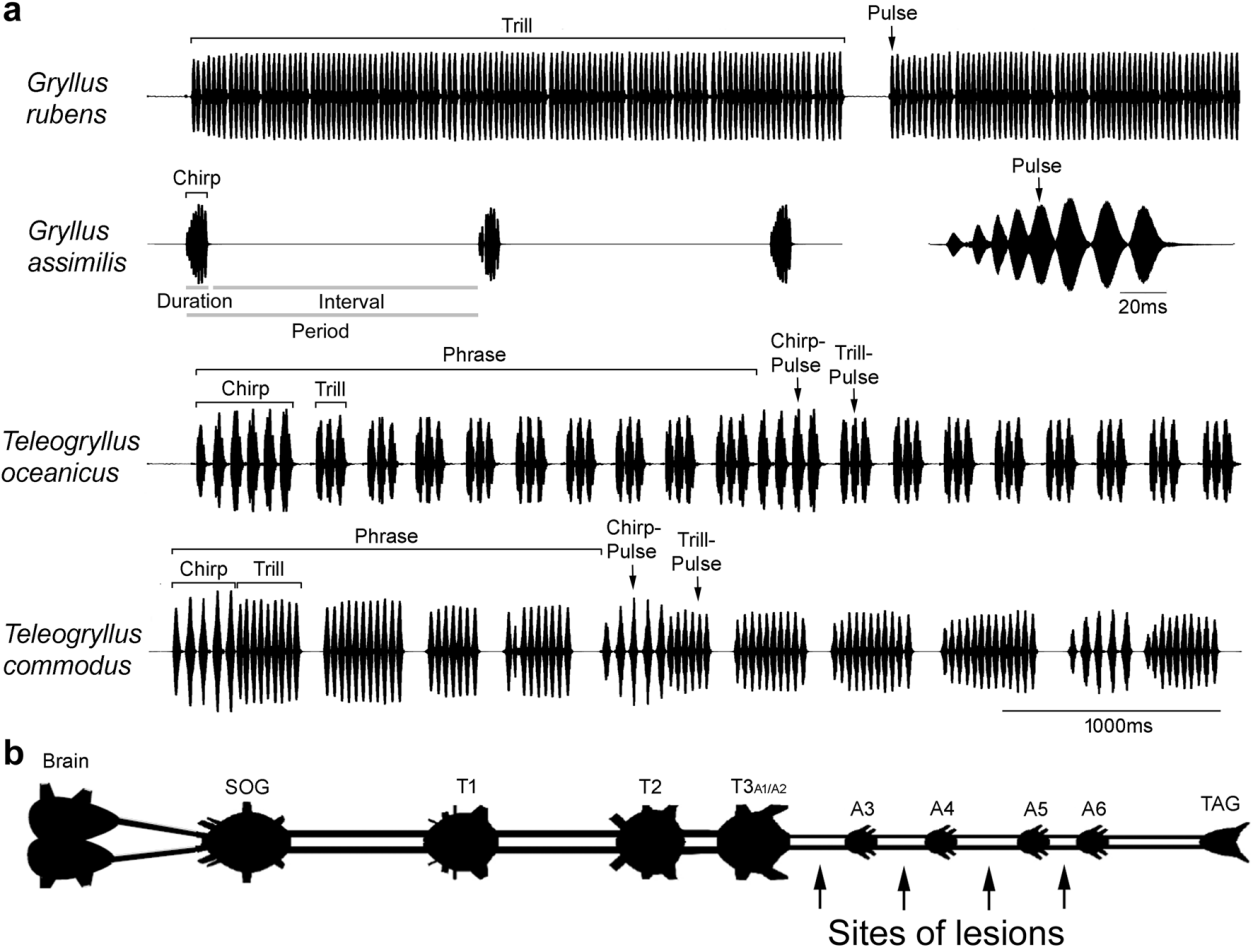
Calling song recordings of four cricket species and diagram of cricket central nervous system. **a** Calling songs of *G. rubens, G. assimilis, T. oceanicus* and *T. commodus* with terminology used for song structure (pulse, chirp, trill, and phrase) and temporal parameters (duration, interval, and period). **b** Schematic representation of a cricket central nervous system and sites of applied lesions, the first 2 abdominal neuromeres are fused with the metathoracic ganglion T3, modified from Jacob and Hedwig (2016)

In preparation to the experiments, male crickets were placed in a 4 °C fridge for 15 min to reduce activity. Immobilised animals were mounted ventral side up on a plasticine block with the body and legs fixed by U-shaped metal hooks. To expose the connectives between specific abdominal ganglia, the appropriate sterna and intersegmental membranes were incised and the wound was temporarily held open by a forceps. Fat body surrounding the target ganglia and connectives was carefully removed while avoiding to damage tracheae and tracheoles. Exposed tissue was covered with insect saline (in mmoll^−1^: NaCl 140; KCl 10; CaCl_2_ 7; NaHCO_3_ 8; MgCl_2_ 1; *N*-trismethyl-2-aminoethanesulfonic acid 5; *D*-trehalose dehydrate 4, pH 7.4) at all times. Lesions were applied with micro scissors to the bilateral connectives between target ganglia. After severing the connectives, the ventral cuticle was folded back, the wound sealed by drying hemolymph, and the insect recovered within one to few days. After several days of sound recording the crickets were sacrificed, and the nervous system was dissected and inspected to confirm the site of the applied lesion. In rare cases that the severed connectives were reconnected by connective tissue, data were treated as invalid and discarded. In crickets the first 2 abdominal neuromeres are fused with the metathoracic ganglion T3. Lesions were applied to the connectives between the metathoracic ganglion complex T3 and the first free abdominal ganglion A3 (T3–A3) and between the posterior abdominal ganglia, i.e. the A3–A4, the A4–A5 and the A5–A6 connectives (Fig. 1b). The connectives between A6 and the terminal ganglion (TAG) were not severed as such lesions do not alter the calling song pattern (Jacob and Hedwig 2016).

### Sound recording

Due to the nocturnal behaviour of crickets, sound recordings were retrieved at night for continuous eight hours. Singing activity was recorded at least for one day before and for one to seven days after applying a lesion. A standard PC microphone (Omni type; Maplin Electronics, Rotherham, UK) was placed in the box of the cricket and sound was recorded with Cool Edit 2000 software (Syntrillium Software Corporation, Phoenix, AZ, USA) running under Windows 7, at a sampling rate of 22 kHz, at 22–24 °C.

### Data analysis and statistics

For each lesion experiment, the songs of at least five animals were recorded and for each animal, recordings of at least 10-min duration before and after the lesion were analysed in terms of song structure and temporal parameters. All analyses of song patterns were carried out with NEUROLAB software (Hedwig and Knepper 1992; Knepper and Hedwig 1997). For songs the duration, interval and period of pulses, chirps, trills and phrases (Fig. 1a) were determined by labeling the start and the end of each pulse, chirp, trill and phrase, respectively. Sound pulses were detected by a threshold algorithm after full-wave rectifying the sound signal. Latency histogram and interval histogram functions were used to calculate the average and standard deviation (X ± SD) for each parameter. The PST-histogram function was applied to calculate the pulse number in chirps or trills, or trill number in phrases.

To define and selectively detect each pulse, a minimum pulse duration was set for each species (*G. assimilis*: 7 ms; *G. rubens*: 20 ms; *T. oceanicus* and *T. commodus*: 30 ms) and visually checked that the start of each pulse was correctly marked. For the description of the calling song structure in *Teleogryllus* species, previous studies have used the terminology of “long chirps” and “short chirps” for the two components of the song of *T. oceanicus, T. emma,* and *T. taiwanemma* (Honda-Sumi 2005; Zuk et al. 2008), while other studies used “chirp” and “trill” in describing the songs of *T. oceanicus* and *T. commodus* (Simmons et al. 2005; Bailey et al. 2017). For consistency, we also use the terms “chirp” and “trill” to describe the two components in *T. oceanicus* and *T. commodus*, furthermore trills in *T. oceanicus* and *T. commodus* may share a similar genetic control (Hoy 1974).

A definition of terms used in this study is given in Fig. 1a and is as follows. *Pulse:* sound pulse as basic unit of cricket songs corresponding to one closing movement of the forewings. *Chirp:* a short sequence with a fixed number of pulses, like a complete song unit in *G. assimilis*, the leading pulse sequence in a song unit in *T. oceanicus* and *T. commodus*. *Trill:* a group of pulses with variable pulse number, i.e. a song unit in *G. rubens*, or a group of pulses with a fixed pulse number, but repeated multiple times after chirps in *T. oceanicus* and *T. commodus*. *Phrase:* a song unit in *Teleogryllus* species consisting of one chirp and several trills. *Sequence:* a group of sound pulses occurring after a lesion in Teleogryllus, with properties between chirp and trill. *Duration:* the time a pulse, chirp, or trill lasts. *Interval:* the time in between pulses, chirps, or trills. *Period:* the time from the start of a pulse, chirp, trill or phrase to the next start of the unit.

For a quantitative analysis of song patterns, 10 min (5 min for *G. rubens*) of recording was selected to create cross-correlograms and raster plots. The cross-correlograms present events in a cumulative way, revealing the frequency distribution of all sound pulses in the selected time window using the first pulse of each chirp, trill, phrase, or sequence as a reference aligned to time zero. In the raster plots, the occurrence of sound pulses was presented and the timing of pulses before and after the reference pulse was plotted over the same time window as used for the cross correlogram. In this way, the timing of pulses of the reference song unit (chirp, trill, phrase, or sequence) and also of pulses of the preceding and subsequent song units (chirp, trill, phrase, or sequence) was displayed. The y-axis position for each row of dots was marginally shifted to a higher value for each new reference event.

In each species, a different time window was required to provide a proper resolution of the timing of sound pulses; we used for *G. rubens*: -5000 to 5000 ms; *G. assimilis*: -50 to 250 ms, − 2000 to 2000 ms, and − 3000 to 3000 ms; *T. oceanicus*: − 500 to 1000 ms; *T. commodus*: − 500 to 2500 ms. To demonstrate the frequency of events, cross-correlograms corresponding to the time window of each raster plot were calculated, using again the first pulse of the chirps, phrases or sequences as reference. The black (control) or light grey (lesion) marked areas in the cross-correlograms indicate the pulse events of the reference chirps, trills, phrases or sequences, respectively, while blue-shaded areas indicate events from previous or subsequent chirps, trills, phrases or sequences, this is not relevant for *G. assimilis.*

Statistical significance of differences in song parameters before and after applying lesions was tested by paired sample t-test applying a two-tailed hypothesis or one-way analysis of variance (ANOVA). Average timing of song parameters in each individual was calculated by NEUROLAB and was exported to SigmaPlot 11.0 (Systat Software, San Jose, CA) for statistical analysis and for drawing bar graphs.

## Results

### Normal calling song patterns of the four cricket species

All males were recorded before a lesion to the abdominal connectives was applied. In the following, an overview of the normal song patterns is given. The calling song of *G. rubens* consists of long trills (Gray and Cade 2000) (Fig. 1a). A total of 284 trills with 24,415 pulses in 13 males were analysed. The trill duration (2170.4 ± 647.4 ms) and trill interval (1361.6 ± 2110.8 ms) were very variable and so was the pulse number within trills (91.2 ± 27.1). On average, pulses in trills had a duration of 13.9 ± 4.3 ms, an interval of 10.5 ± 6.1 ms, and a period of 23.9 ± 2.6 ms.

The calling song of *G. assimilis* contains chirps repeated at rather long intervals (Pollack and Kim 2013) (Fig. 1a). Analysis of 4742 chirps with a total of 34,158 pulses in 11 males showed that one chirp contained six to nine sound pulses (7.4 ± 0.5 pulses). In this species, some chirps showed clear pulse interval while other chirps showed no or only a very short pulse interval (Fig. 1a and Fig. 4a). We therefore report only the pulse period (14.0 ± 2.8 ms). The chirps of *G. assimilis* had an average duration of 93.5 ± 16.3 ms, while the chirp intervals were long and come with high variability (1447.6 ± 772.4 ms).

The calling song of *T. oceanicus* contains phrases composed of one chirp and several trills (Zuk et al. 2008; Bailey et al. 2017) (Fig. 1a). In 10 males, 401 phrases containing 401 chirps with 1855 chirp-pulses, and 3008 trills with 6597 trill-pulses were recorded and analysed. The phrase period was 1606.8 ± 395.2 ms, chirp duration was 283.4 ± 62.8 ms, trill duration was 80.3 ± 23.3 ms, trill interval was 74.5 ± 15.5 ms, and trill period was 153.3 ± 17.3 ms. Each phrase contained 8.1 ± 2.4 trills on average. In chirps, the pulse duration was 35.3 ± 7.4 ms and similar in length to the pulse interval (31.2 ± 10.8 ms), while in trills the pulse duration was 28.8 ± 7.1 ms and was considerably longer than the pulse interval (13.0 ± 7.4 ms). The difference in chirps and trills is indicated by the pulse period which was 66.5 ± 5.3 ms in chirps and 41.6 ± 5.8 ms in trills, therefore in the oscillogram chirp-pulses appear to be more loosely packed than trill-pulses. On average, chirps contained 4.7 ± 0.6 pulses and trills contained two or three pulses (2.2 ± 0.4 pulses), but occasionally more than 3 pulses could occur.

In *T. commodus*, the calling song is composed of phrases with one chirp followed by a few trills (Simmons et al. 2005; Bailey et al. 2017) (Fig. 1a). A total of 1234 phrases containing 1234 chirps with 6929 chirp-pulses and 2606 trills with a total of 31,087 trill-pulses in 10 males were analysed. The phrase period was 1822.8 ± 509.6 ms, chirp duration was 337.7 ± 62.6 ms, trill duration was 553.6 ± 431.6 ms, trill interval was 236.4 ± 130.7 ms, and trill period was 824.6 ± 539.1 ms. Each phrase includes one chirp and 2.2 ± 1.1 trills. Similar to *T. oceanicus*, chirps in *T. commodus* had a pulse duration of 31.5 ± 3.4 ms and a pulse interval of 30.6 ± 4.6 ms; whereas trills had a longer pulse duration (25.4 ± 3.8 ms) than the pulse interval (13.0 ± 2.7 ms). This is reflected in the difference of the chirp-pulse period (61.9 ± 5.5 ms) and the trill-pulse period (38.4 ± 3.9 ms), and in the dense pattern of trill-pulses in the oscillogram. Each chirp contained 5.5 ± 1.0 pulses and each trill contained 14.7 ± 10.2 pulses on average.

### General effects after lesions

A total of 164 male crickets (42 *G. assimilis*, 30 *G. rubens*, 51 *T. oceanicus*, and 41 *T. commodus*) were used in this study. Seven males died within three days after applying a lesion (5 *G. assimilis* and 2 *T. commodus*). In these males, severe reduction of mobility and a constantly low position of the antennae was observed. 32 males (8 *G. assimilis*, 3 *G*. *rubens*, 13 *T. oceanicus*, and 8 *T. commodus*) never sang again after a lesion, even some of them lived for another three weeks. After a lesion to the abdominal nerve cord all males no longer responded with escape behaviour to stimulating the cerci with a pen brush, they failed to copulate and many showed an accumulation of feces at their anus. The rest of the cohort did not show any obvious defects in walking and ingestion, and their post-lesion calling songs were recorded successfully. Life span after lesion ranged from a week to two months (68 days the longest). In all four species, the impact of the T3–A3 lesion and of the A3–A4 lesions, respectively, was very similar, these data will be presented together. Lesions of A4–A5 and A5–A6 connectives had more specific effects and will be presented separately for each species.

### All species lose calling song activity after lesions to the T3–A3 connectives

**Fig. 2:**
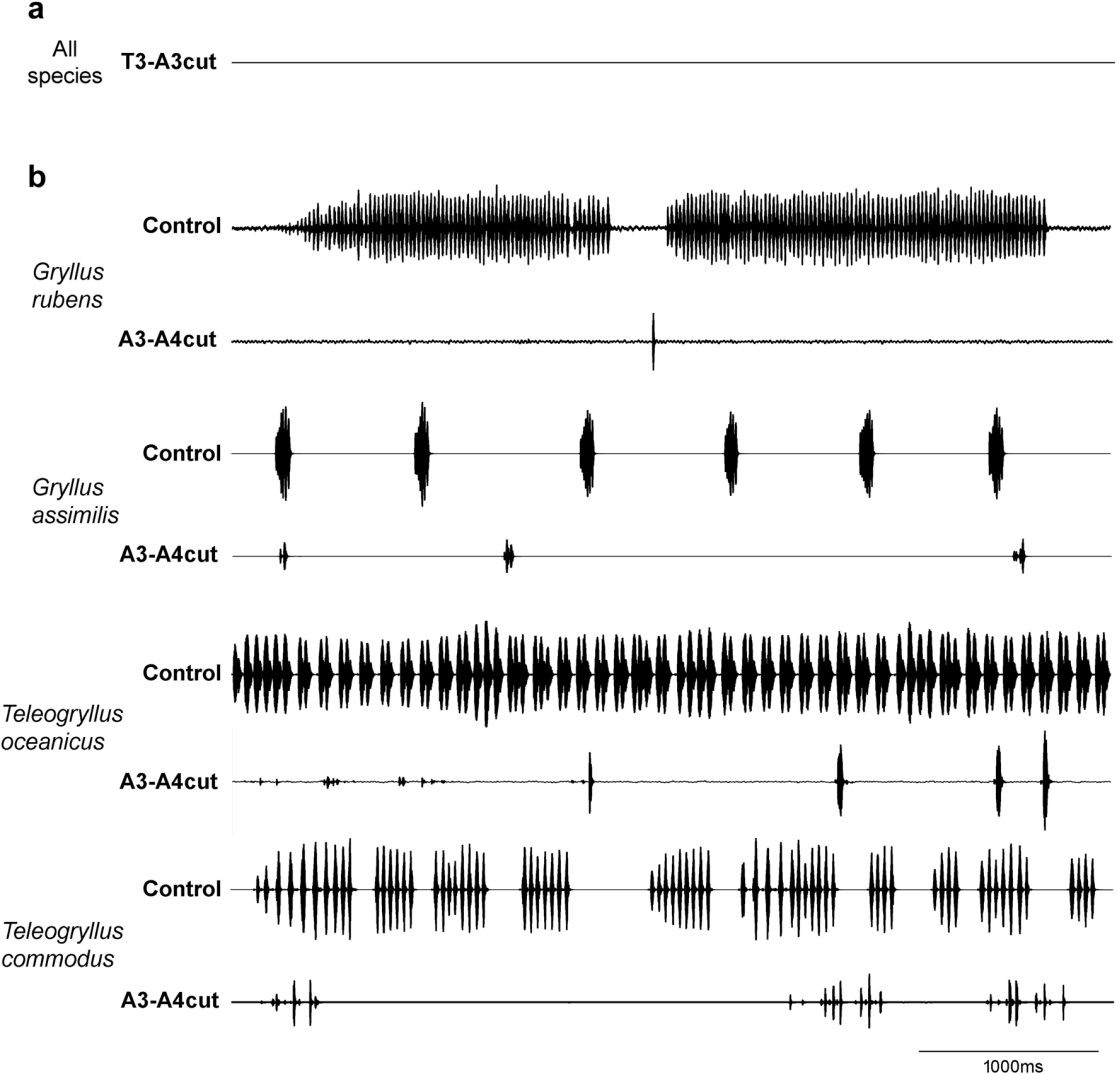
Representative sound recording of *G. rubens, G. assimilis, T. oceanicus* and *T. commodus* for males with an T3-A3 or A3-A4 lesion. **a** Recording indicates failure of calling song production in the four cricket species after T3-A3 lesion. **b** Calling song recordings before and after an A3-A4 lesion in *G. rubens, G. assimilis, T. oceanicus* and *T. commodus*

In crickets with their connective cut between the metathoracic ganglion complex T3 and the first free abdominal ganglion A3, the sound recordings did not demonstrate any sign of singing activity. Without exceptions, all the males of the four species (T3–A3cut: 5 *G. rubens*, 6 *G. assimilis*, 8 *T. oceanicus*, and 5 *T. commodus*) no longer produced identifiable sound pulses once the T3–A3 connectives were severed (Fig. 2a). Males still raised their forewings into singing position, but no coordinated opening-closing movements of the forewings ever occurred while watching the animals for extended periods of time. Most males with a T3–A3 lesion lived for another one to two weeks, they showed no deficiency in locomotor activity before they died.

### All species lose the calling song structure after lesions to the A3–A4 connectives and generate single pulses only

All four cricket species showed similar effects on the calling song structure after the connectives between A3 and A4 were sectioned (A3-A4cut: 7 *G. rubens*, *6 G. assimilis*, 9 *T. oceanicus*, and 6 *T. commodus*). Overall, these males strongly reduced their singing activity and generated less than 100 sound pulses in an overnight recording (*G. rubens*: 37.1 ± 36.2 pulses, n = 7; *G. assimilis*: 58.7 ± 19.7 pulses, n = 6; *T. oceanicus*: 83.9 ± 113.7 pulses, n = 9; *T. commodus*: 26.7 ± 21.7 pulses, n = 6). These males either produced “scratchy” sound of short duration and low amplitude, or single pulses only without a higher song structure (Fig. 2b). The pulses, due to quivering or lack of proper engagement of the wing movements, were usually incomplete and clearly lower in sound amplitude (Jacob and Hedwig 2016) but in the same frequency range as normal pulses. One *G. assimilis* male produced only single pulses for the first week, after which it began to sing normal calling songs. Dissection of this animal revealed a tissue connection of the separated connectives, either due to an incomplete lesion or a possible regrowth.

## Effects of lesions in *Gryllus rubens*

### No change in calling song pattern after lesion to the A4–A5 connectives in *Gryllus rubens*

**Fig. 3:**
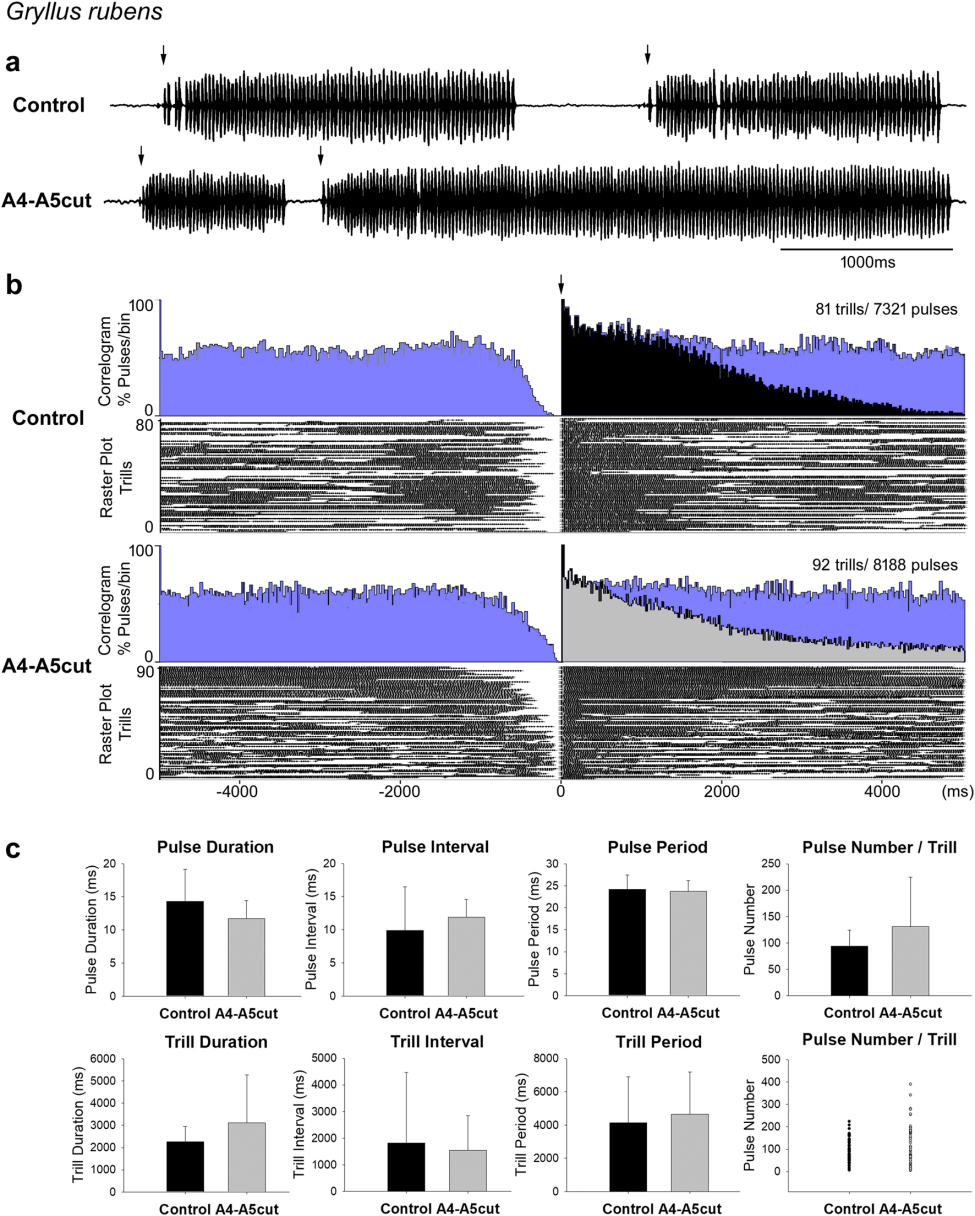
Calling song before and after the A4-A5 lesion in *G. rubens*. ** a** Song recording before (top) and after the lesion (bottom). Arrows indicate the start of each trill. **b** Cross-correlogram and raster plot of 5-min song recording of one male before and after the lesion on a time scale from -5000 ms to 5000 ms. In correlograms, black (control) and light grey (A4-A5cut) shading represent the pulses of the reference-trills and blue shading indicates the pulses of the subsequent (to the right) or previous (to the left) trill. Arrow indicates the start of trills, taken as reference. **c** Temporal parameters of calling song before and after the lesion in 8 males

Male *G. rubens* exhibited normal singing activity after an A4–A5 lesion was applied and produced normal trills (A4–A5 cut, Fig. 3a). Cross-correlogram and raster plot were drawn based on a 5-min recording of one male (Fig. 3b). In the correlograms, black (control, top) and light grey (A4–A5cut, bottom) denote the pulses of the reference trills, respectively. Blue denotes the pulses of previous or subsequent trills. In both groups, pulse number, trill duration, and trill interval were variable. Because of the variable pulse number and trill duration, the frequency of pulses in reference-trills gradually declines with increasing time (black and light grey shade in correlogram), while the frequency of pulses in the subsequent trill gradually increases (blue shade).

Calling songs of 8 males before (155 trills, 14,349 pulses) and after an A4-A5 lesion (177 trills, 15,625 pulses) were recorded and analysed. As trills produced by intact males were already variable in terms of trill parameters, there was no significant difference between the control and operated group in respect to trill duration (control: 2260.2 ± 696.2 ms, A4-A5cut: 3104.6 ± 2157.2 ms, *p* = 0.310), trill interval (control: 1814.0 ± 2647.5 ms, A4-A5cut: 1544.8 ± 1292.0 ms, *p* = 0.800), trill period (control: 4147.3 ± 2746.0 ms, A4-A5cut: 4643.9 ± 2553.7, *p* = 0.714), and pulse number in trills (control: 94.2 ± 29.6 pulses/trill, A4-A5cut: 131.1 ± 93.3 pulses/trill, *p* = 0.304) (Fig. 3c). Although in one male, some trills showed substantially more pulses after the A4-A5 lesion, this was not observed in other males and overall this did not lead to a significant difference in the mean pulse number of trills (Fig. 3c, Pulse Number/Trill, dot graph).

Pulse parameters in A4-A5cut males also showed no significant difference compared to intact males (Fig. 3c). Pulse duration was 14.3 ± 4.8 ms in the control group and 11.7 ± 2.7 ms in the A4-A5cut group (*p* = 0.206). Pulse interval was 9.9 ± 6.69 ms in the control group and 11.9 ± 2.7 ms in the A4-A5cut group (*p* = 0.439). Pulse period was similar between control (24.2 ± 3.3 ms) and A4-A5cut males (23.8 ± 2.4 ms, *p* = 0.780).

Overall, calling song of *G. rubens* contained trills with variable temporal parameters and no significant change occurred after the A4-A5 lesion was applied.

### No change in calling song pattern after lesions to the A5-A6 connectives in *Gryllus rubens*

As expected from the outcome of the previous experiment, male *G. rubens* generated normal trills after the connectives between A5 and A6 were severed.

Like intact animals, A5-A6cut males showed a substantial variation in the trill parameters. In 5 males, 136 trills (9787 pulses) under intact condition and 107 trills (8790 pulses) after the A5-A6 lesion were analysed. There was no significant difference between the control and the A5-A6 group in terms of trill duration (control: 2026.8 ± 605.9 ms, A5-A6cut: 1723.7 ± 553.4 ms, *p* = 0.433), trill interval (control: 637.8 ± 187.2 ms, A5-A6cut: 488.3 ± 210.2 ms, *p* = 0.269), trill period (control: 2622.4 ± 620.7 ms, A5-A6cut: 2203.9 ± 510.3 ms, *p* = 0.278), and pulse number in trills (control: 86.6 ± 24.9 pulses/trill, A5-A6cut: 76.6 ± 26.6 pulses/trill, *p* = 0.558). Even though few trills showed a higher number of pulses/trill in the A5-A6cut animals, the overall pulse number was not significantly different from intact animals.

Also regarding pulse parameters, there was no significant difference in pulse duration (control: 13.1 ± 3.6 ms, A5-A6cut: 11.4 ± 4.4 ms, *p* = 0.518), pulse interval (control: 10.2 ± 3.1 ms, A5-A6cut: 11.4 ± 5.9 ms, *p* = 0.685), or pulse period (control: 23.4 ± 0.8 ms, A5-A6cut: 22.8 ± 2.5 ms, *p* = 0.626) between intact males and A5-A6cut males.

In summary, calling songs of *G. rubens* showed no significant change in song parameters before and after an A5-A6 lesion, as trills with a variable pulse number, duration, and interval were generated by the males. This is in line with the A4-A5 lesions, which did not cause significant changes.

## Effects of lesions in *Gryllus assimilis*

### Extended chirps occur after lesions to the A4-A5 connectives in *Gryllus assimilis*

**Fig. 4:**
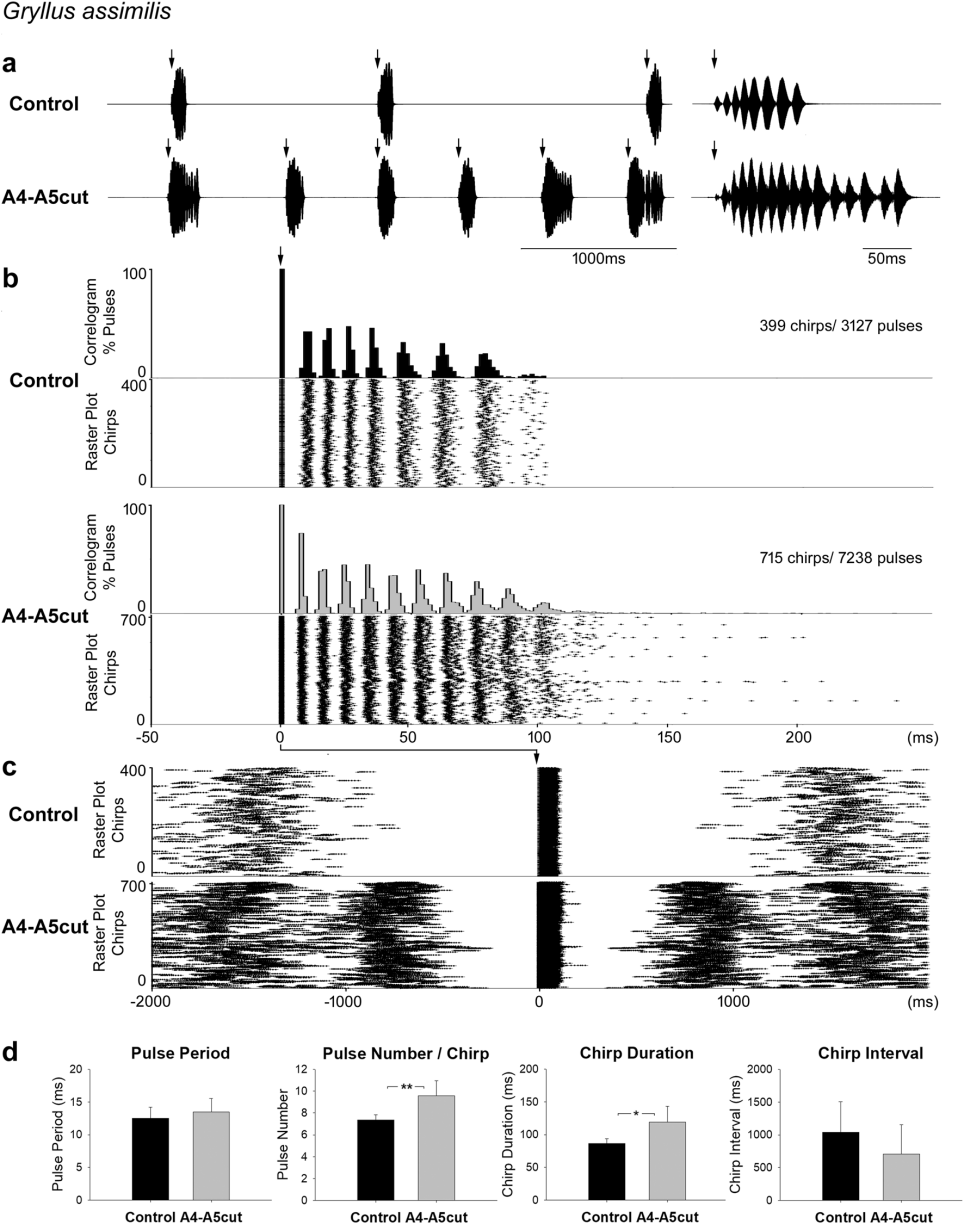
Calling song before and after the A4-A5 lesion in *G. assimilis.* **a** Song recording before (top) and after the lesion (bottom). The first chirp of the section is shown in high resolution on the right hand side. Arrows indicate the start of each chirp. **b** Cross-correlogram and raster plot of a 10-min calling song recording of one male before (top) and after the lesion (bottom) over a time scale from − 50 ms to 250 ms. Arrow indicates the start and first pulse of chirps, taken as reference and aligned to 0 ms, all subsequent pulses give rise to separate peaks in the correlograms with increasing scatter. The raster plot shows the timing of the sound pulses in the vertical rows of dots. Each dot represents one sound pulse, chirps are indicated by sequences of horizontal dots. **c** Raster plot for the same recordings before and after the lesion over a time scale from − 2000 ms to 2000 ms. Arrow indicates the start of chirps, taken as reference. Dots to the left and right end of the raster plot indicate the sound pulses of previous and subsequent chirps, respectively. **d** Temporal parameters of the calling song before and after the lesion in 6 males (* = *p* < 0.05, ** = *p* < 0.01)

*G. assimilis* males showed continued singing activity after an A4-A5 or A5-A6 lesion was applied. They produced calling songs with extended chirps after the connectives between A4 and A5 were incised (A4-A5cut, Fig. 4a).

Cross-correlogram and raster plot based on a 10-min calling song recording of one male demonstrate the change. Before the A4-A5 lesion, calling song chirps contained up to nine pulses (Fig. 4b). After the lesion, the pulse number in chirps increased and became more variable, the raster plot shows chirps containing ten pulses while more than 20 pulses occurred in some other chirps. The longest chirp produced after an A4–A5 lesion contained over 30 pulses. At higher time resolution (Fig. 4c), the raster plot reveals the shorter chirp interval after the A4-A5 lesion as more chirps occur in the given time frame. This is also demonstrated by more chirps generated in the 10-min recording after the A4–A5 lesion as compared to the control (control: 399 chirps, A4–A5cut: 715 chirps). However, not all the males showed a reduced chirp interval after the lesion.

Calling songs of 6 males before (2993 chirps, 21,644 pulses) and after the lesion (4799 chirps, 43,703 pulses) were analysed. Chirps after the A4-A5 lesion had significant more pulses (9.6 ± 1.4 pulses/chirp, *p* = 0.005) and showed a longer chirp duration (119.2 ± 24.2 ms, *p* = 0.01) compared to chirps produced before the operation (7.4 ± 0.5 pulses/chirp and 86.3 ± 7.2 ms) (Fig. 4a, d). Though not significant (*p* = 0.239), after the A4-A5 lesion 5 of 6 males showed shorter chirp intervals (708.4 ± 448.7 ms) than chirps produced by the intact males (1039.9 ± 467.6 ms) (Fig. 4d). The pulse period was not significantly different between the two groups (control: 12.5 ± 1.7 ms and A4-A5cut: 13.5 ± 2.1 ms, *p* = 0.398). Overall, the A4-A5 lesion increased the pulse number in chirps and the chirp pattern showed a tendency of a reduced chirp interval, but the lesion did not alter the pulse period.

## No change in calling song pattern after lesions to the A5-A6 connectives in *Gryllus assimilis*

*Gryllus assimilis* produced calling song with normal chirps after the connectives between A5 and A6 were severed. Calling songs of 5 males before (1749 chirps, 12,514 pulses) and after the lesion (2262 chirps, 16,799 pulses) were analysed. The calling songs after the lesion were similar to the normal calling song and showed no significant difference in temporal parameters. Pulse period of A5-A6cut males (13.9 ± 1.6 ms) was close to the pulse period in control recordings (15.7 ± 3.0 ms, *p* = 0.282). Pulse number in chirps did not change before (7.4 ± 0.5 pulses/chirp) and after the lesion (7.5 ± 0.37 pulses/chirp, *p* = 0.715). In terms of chirp parameters, chirp duration after the lesion (90.9 ± 14.0 ms) was similar to normal calling song (102.1 ± 20.8 ms, *p* = 0.349). There was no significant difference between chirp intervals before (1936.8 ± 818.3 ms) and after the lesion (1248.0 ± 365.1 ms, *p* = 0.124), even 4 of 5 operated males showed a shorter chirp interval. In summary the A5-A6 lesion did not change the temporal parameters of the song structure.

## Effects of lesions in *Teleogryllus oceanicus*

### The phrase structure is lost after lesions to the A4-A5 connectives in *Teleogryllus oceanicus*

**Fig. 5:**
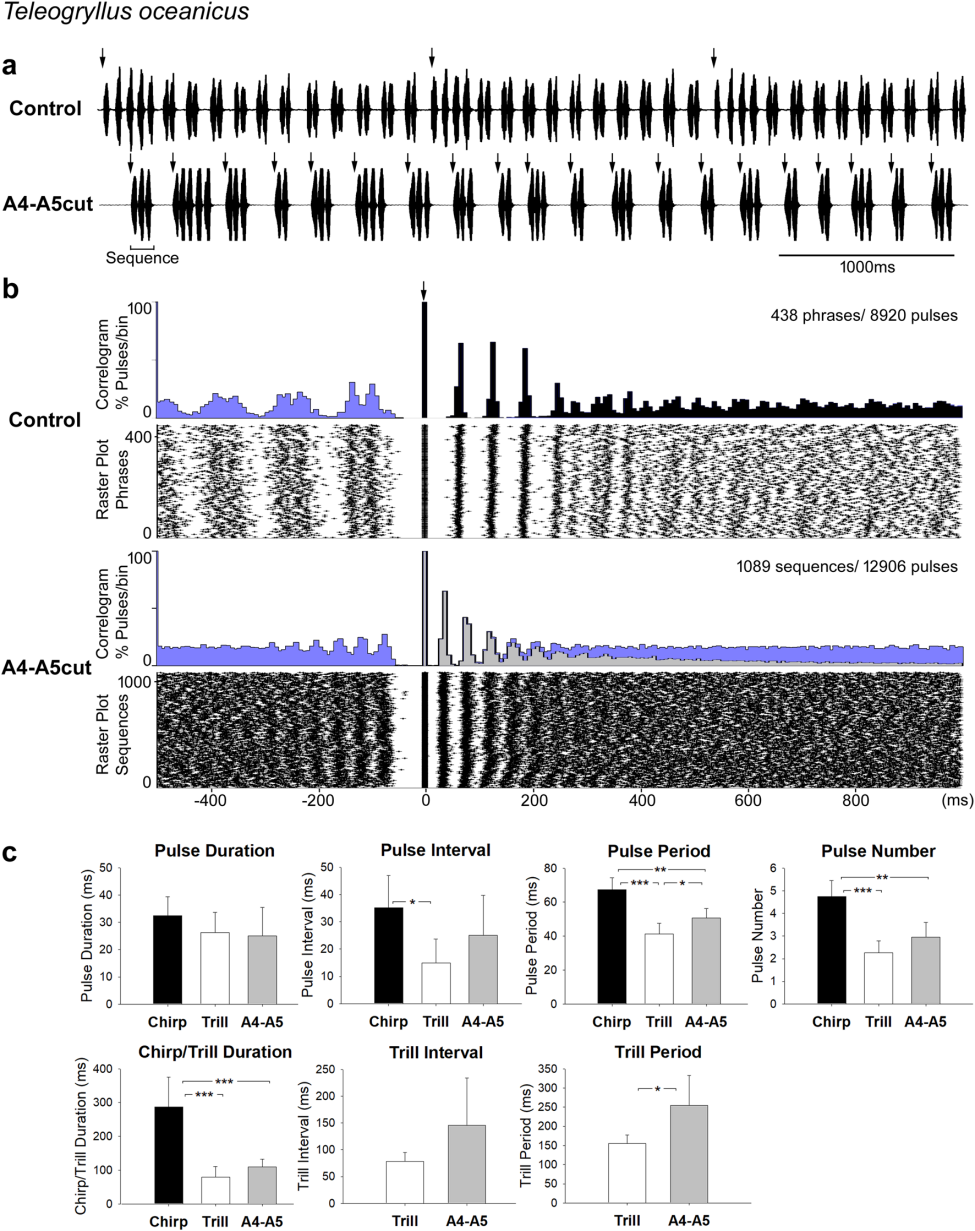
Calling song before and after the A4-A5 lesion in *T. oceanicus.* **a** Song before (top) and after the lesion (bottom). Arrows indicate the start of each phrase or sequence used as reference in the data analysis. **b** Cross-correlogram and raster plot of 10-min song recordings of one male before and after the lesion at a time scale from − 500 ms to 1000 ms. Black (control) and light grey (A4-A5cut) shading represent the reference phrases or reference sequences in correlograms and blue shading indicates the pulses of the subsequent (to the right) or previous (to the left) phrase or sequence. Arrow indicates the start of reference phrases and reference sequences, respectively. **c** Temporal parameters of the calling song before and after the lesion in 5 males (* = *p* < 0.05, ** = *p* < 0.01, *** = *p* < 0.001)

Male *T. oceanicus* continued to sing regularly after the connectives between A4 and A5, or between A5 and A6, were cut. The calling song after the A4-A5 lesion showed substantial changes of the song structure. The two song components in the phrases, the chirps and trills, were no longer generated and were replaced by a repetition of groups of sound pulses, which were intermediate between chirps and trills and here are called *sequences* (Fig. 5a). Without the intermittent onset of the two song components, the calling song generated by A4-A5cut males sounded more like the pattern of chirp-producing species.

The cross correlogram and raster plot of 10-min song recording before and after the A4-A5 lesion in one male highlight the change in song structure (Fig. 5b). In the control recording, 4 chirp-pulses with longer pulse intervals occur at the start of each phrase between 0 and 200 ms, giving rise to four peaks in the correlogram, corresponding to the four vertical bands of dots indicating the chirp-pulses in the raster plot. Trill-pulses occurred before and after the chirp-pulses, the trill-pulses before 0 ms showed the typical doublet pattern of trill-pulses giving rise to bands of two coupled pulses in the raster plot and two corresponding peaks in correlograms, e.g. between − 150 ms and − 100 ms. The scatter for the timing of the trill-pulses increased with increasing the interval before the reference-phrase. The trill-pulses occurring after the chirps are reflected in the flat part of the correlogram from 300 to 1000 ms, and in the corresponding distribution of dots in the raster plot. The clear pattern of trills as seen in the control sound recording is gradually lost as the timing of the trill-pulses occurs with an increasing scatter in relation to the start of the reference-chirp and also as doublets and triplets of trill-pulses occurred after chirps.

In the A4–A5 lesion group, chirps and trills were no longer obvious, rather 2 to 4 pulses were grouped in sequences. There was only one form of sequence-pulses, it occurred with a shorter interval as chirp-pulses and a longer interval as trill-pulses in intact animals. The timing of the sound pulses was analyzed with the first pulse of each sequence taken as reference. The correlograms and raster plot reveal the characteristic temporal organization of the reference-sequences. However, with increasing time intervals, the timing of subsequent sequences becomes scattered, relative to the start of the reference-sequences, because of the variable sequence durations. Looking however, at the events before the reference-sequences reveals that the interval between sequences was very stable and similar like in the normal song.

In 5 males, 945 phrases (945 chirps, 4352 chirp-pulses, 7674 trills, 17,073 trill-pulses) before the lesion and 5925 sequences with 17,929 sequence-pulses after the lesion were analysed. To evaluate the change in the song structure caused by the A4-A5 lesion, pulse parameters of original chirps and trills, and of the sequences were compared (Fig. 5c).

Most pulse parameters of sequences were between the values of normal chirps and trills: pulse interval (chirp: 35.2 ± 11.8 ms, trill: 15.0 ± 8.7 ms, sequence: 25.1 ± 14.6 ms, P(chirp vs trill) = 0.015, P(chirp vs sequence) = 0.263, P(trill vs sequence) = 0.222); pulse period (chirp: 67.4 ± 6.9 ms, trill: 41.4 ± 6.2 ms, sequence: 50.7 ± 5.6 ms, P(chirp vs trill) < 0.001, P(chirp vs sequence) = 0.003, P(trill vs sequence) = 0.037), and pulse number (chirp: 4.7 ± 0.7 pulses, trill: 2.3 ± 0.5 pulses, sequence: 3.0 ± 0.6 pulses, P(chirp vs trill) < 0.001, P(chirp vs sequence) = 0.008, P(trill vs sequence) = 0.096). However the sequence-pulse duration remained closer to the duration of pulses in trills, (chirp: 32.5 ± 6.9 ms, trill: 26.2 ± 7.4 ms, sequence: 25.0 ± 10.4 ms, P(chirp vs trill) = 0.207, P(chirp vs sequence) = 0.220, P(trill vs sequence) = 0.840).

In terms of song structure, the sequence duration (109.4 ± 23.1 ms) was closer to the trill duration (79.3 ± 31.2 ms) than to chirp duration (286.9 ± 88.3 ms, P(chirp vs trill) < 0.001, P(chirp vs sequence) < 0.001, P(trill vs sequence) = 0.121). Sequences however showed longer intervals and thus had longer periods than the trills (sequence interval: 145.7 ± 88.5 ms, trill interval: 78.3 ± 16.9 ms, *p* = 0.133; sequence period: 254.3 ± 78.0 ms, trill period: 155.7 ± 22.1 ms, *p* = 0.026).

As a consequence of the A4-A5 lesion the normal chirp and trill structure of phrases found in *T. oceanicus* calling song was abolished, and the resulting song pattern consisted of short intermediate sequences of 2–4 pulses with an intermediate pulse structure.

## No change in calling song pattern after lesions to the A5-A6 connectives in *Teleogryllus oceanicus*

**Fig. 6:**
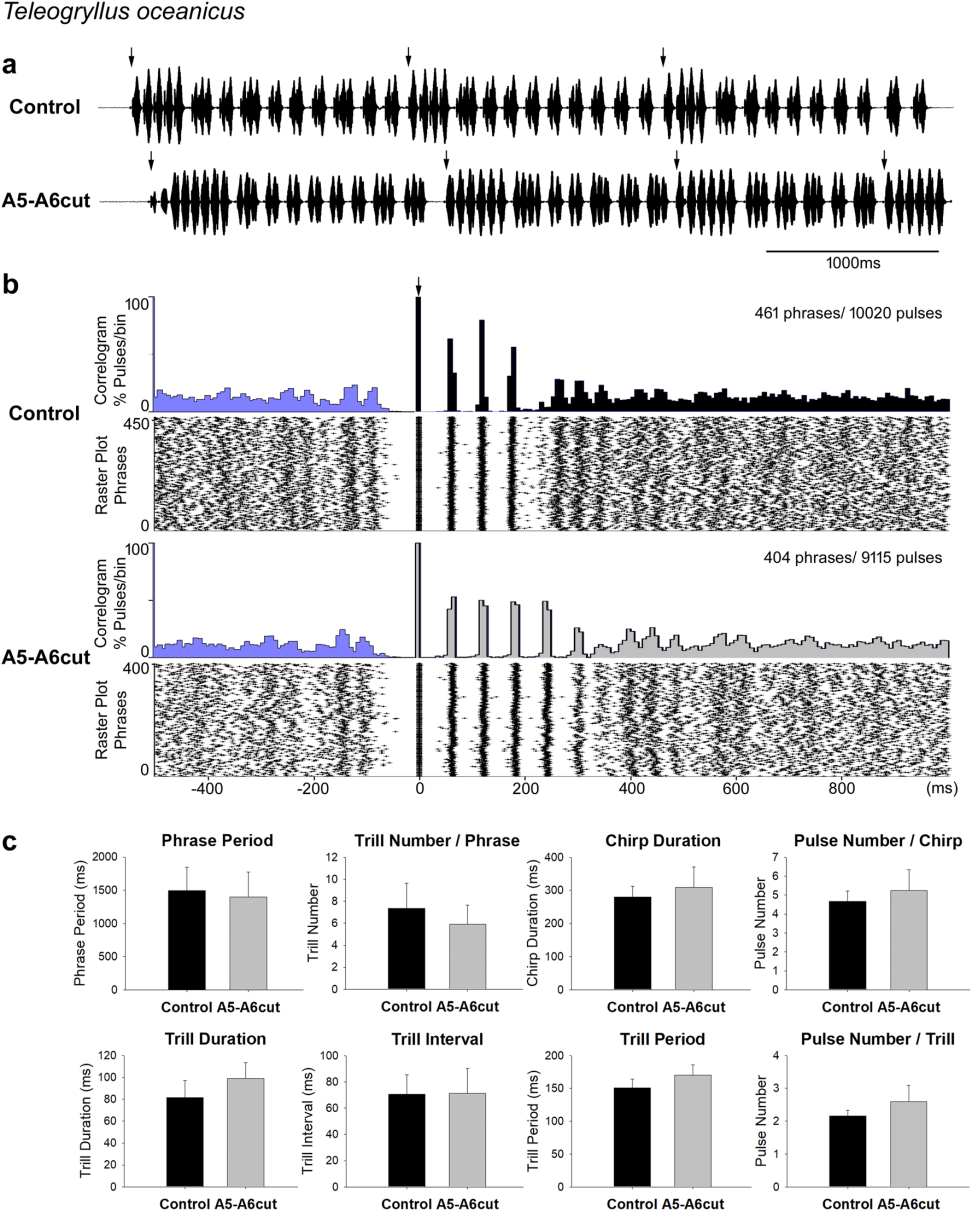
Calling song before and after the A5-A6 lesion in *T. oceanicus*. **a** Song before (top) and after the lesion (bottom). Arrows indicate start of each phrase, used as reference for the calculation of correlograms and raster plots. **b** Cross-correlogram and raster plot of 10-min song recordings of one male before and after the lesion over a time scale from -500 ms to 1000 ms. Black (control) and light grey (A5-A6cut) shade represent the reference-phrases in correlograms and blue shading indicates the pulses of the subsequent (to the right) or previous phrases (to the left). Arrow indicates the start of phrases. **c** Temporal parameters of calling song before and after the lesion in 5 males No significant differences occurred between the intact and A5-A6cut group

Calling song generated by *T. oceanicus* after the connectives between A5 and A6 were severed showed phrases with chirps and trills as the calling song in intact males. Each phrase was composed of one chirp followed by several trills, which consisted of doublet or triplet sound pulses (Fig. 6a). Two 10-min song recordings of one male before and after the A5-A6 lesion were used to generate cross-correlograms and raster plots (Fig. 6b). In both groups, chirp-pulses showed as pronounced and sharp peaks in the correlogram and were properly aligned to the raster plot; there were 4 peaks for chirp-pulses under intact condition and 5 peaks in the A5-A6cut condition. Chirp-pulses had longer pulse intervals than trill-pulses, which followed subsequent to the chirp-pulses. As the occurrence of the trill-pulses was plotted relative to the start of the chirps, and due to the variability of the pulse number in trills, the scatter of the trill-pulses gradually increased and only the trill-pulses following the chirp between 250 and 350 ms showed clear peaks in the correlogram. The three vertical bands in the raster plot and the three peaks in the correlogram suggest these trills were triplets, while the following trills could be doublets or triplets of pulses. In the A5-A6cut recording, there were signs of triplets at 400 ms and doublets at 600 ms. For both recordings, the trills preceding the reference chirps showed doublet pulses between − 150 ms and − 100 ms and revealed that the interval between the end of the last trill in a phrase and the subsequent chirp remained very similar in the control and lesioned male.

In 5 males, 1060 phrases (1060 chirps, 4925 chirp-pulses, 7367 trills, and 15,911 trill-pulses) before the lesion and 1120 phrases (1120 chirps, 5936 chirp-pulses, 6345 trills, and 16,441 trill-pulses) after the lesion were analysed regarding the temporal parameters. There was no significant difference between the two groups in respect to phrase period (control: 1492.3 ± 352.9 ms, A5-A6cut: 1396.8 ± 375.5 ms, *p* = 0.689), trill number in phrases (control: 7.4 ± 2.3 trills/phrase, A5-A6cut: 5.9 ± 1.7 trills/phrase, *p* = 0.292), chirp duration (control: 279.8 ± 32.3 ms, A5-A6cut: 309.2 ± 61.9 ms, *p* = 0.374), pulse number in chirps (control: 4.7 ± 0.6 pulses/chirp, A5-A6cut: 5.2 ± 1.1 pulses/chirp, *p* = 0.321), trill duration (control: 81.4 ± 15.74 ms, A5-A6cut: 98.94 ± 14.54 ms, *p* = 0.103), trill interval (control: 70.74 ± 14.74 ms, A5-A6cut: 71.14 ± 19.1 ms, *p* = 0.972), trill period (control: 150.9 ± 13.1 ms, A5-A6cut: 170.2 ± 16.0 ms, *p* = 0.071), and pulse number in trills (control: 2.2 ± 0.2 pulses/trill, A5-A6cut: 2.6 ± 0.5 pulses/trill, *p* = 0.091), (Fig. 6c).

Also the pulse parameters of chirps and trills after the A5-A6 lesion were not significantly different from the songs of intact males. For chirps, the chirp-pulse duration (control: 38.2 ± 7.3 ms, A5-A6cut: 37.9 ± 8.3 ms, *p* = 0.953), chirp-pulse interval (control: 27.3 ± 9.2 ms, A5-A6cut: 24.7 ± 10.0 ms, *p* = 0.686), and chirp-pulse period (control: 65.6 ± 3.6 ms, A5-A6cut: 62.7 ± 3.7 ms, *p* = 0.240) are not different between the two groups. The trill-pulse parameters like trill-pulse duration (control: 31.3 ± 6.5 ms, A5-A6cut: 31.4 ± 6.1 ms, *p* = 0.972), trill-pulse interval (control: 11.0 ± 6.2 ms, A5-A6cut: 10.7 ± 6.1 ms, *p* = 0.944), and trill-pulse period (control: 41.9 ± 6.1 ms, A5-A6cut: 41.7 ± 2.3 ms, *p* = 0.956) also showed no significant difference before and after the lesion.

Overall, the calling song generated by *T. oceanicus* males with an A5-A6 lesion had not changed in terms of the phrase structure and the parameters of the pulse patterns.

## Effects in *Teleogryllus commodus*

### The phrase structure is lost after lesions to the A4-A5 connectives in *Teleogryllus commodus*

**Fig. 7:**
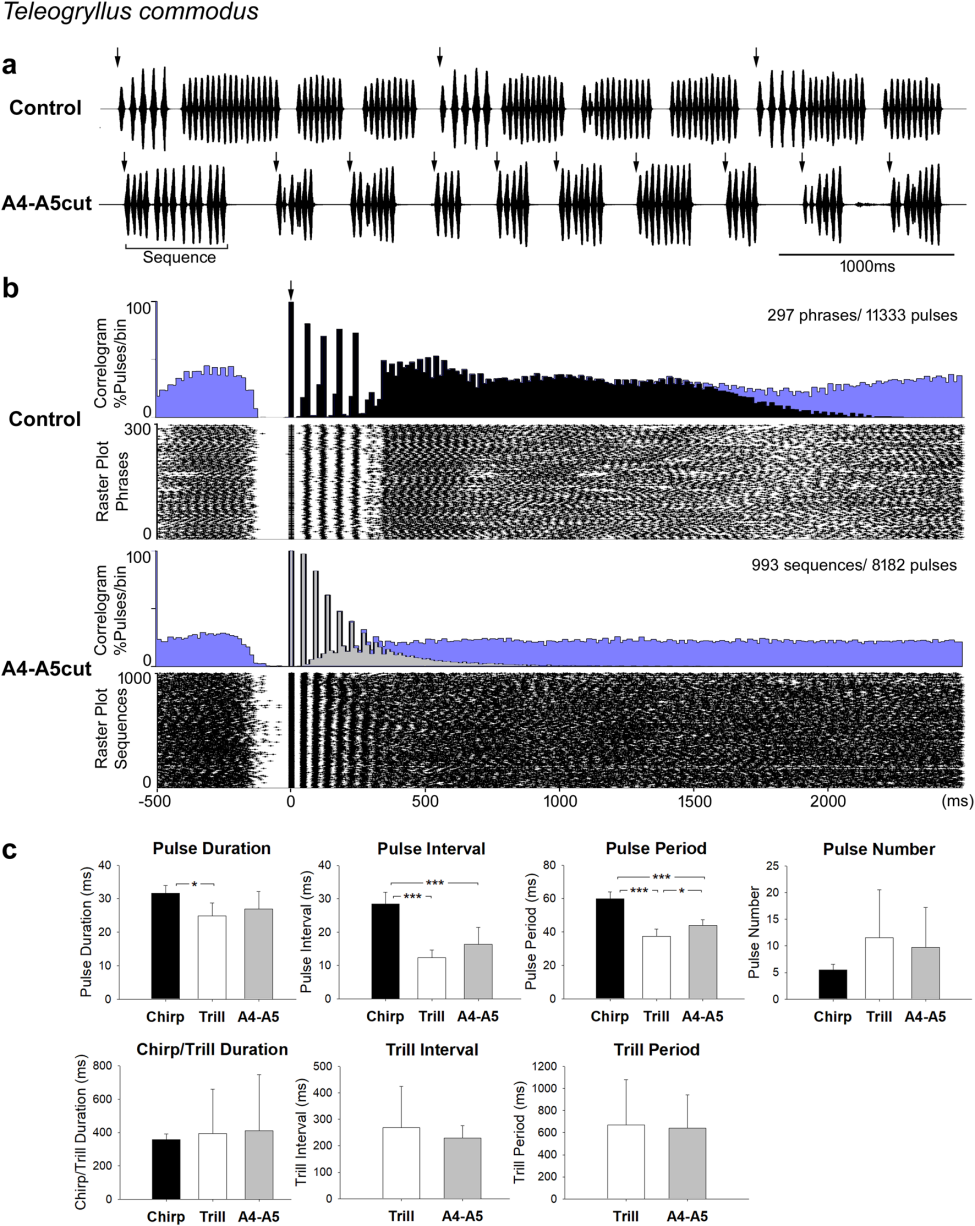
Calling song before and after the A4-A5 lesion in *T. commodus.* **a** Song recording before (top) and after the lesion (bottom). Arrows indicate start of each phrase or sequence, taken as reference for the histograms. **b** Cross-correlogram and raster plot of 10-min song recording of one male before and after the lesion over a time scale from -500 ms to 2500 ms. Black (control) and light grey shade (A4-A5cut) represent reference phrases or reference sequences in correlograms and blue shading indicates the pulses of the subsequent (to the right) or previous (to the left) phrases or sequences. Arrow indicates the start of phrases and sequences. **c** Temporal parameters of calling song before and after lesion in 5 males. (* = *p* < 0.05, ** = *p* < 0.01, *** = *p* < 0.001)

Male *T. commodus* continued to sing after connectives between A4 and A5, or A5 and A6, were lesioned. Lesions to the connectives between A4 and A5 had a similar effect on the calling song of *T. commodus* as in *T. oceanicus*. Phrases consisting of chirps and trills were no longer generated by A4-A5cut males, and were replaced by repeated short groups of sound pulses, called sequences (Fig. 7a). These songs composed of sequences sounded like calling song from a chirp-producing species, but with a variable pulse number.

Cross-correlogram and raster plot based on 10-min song recordings of one male show the change of song structure after the A4-A5 lesion (Fig. 7b). For the control group, five peaks in the correlogram and five aligned bands in the raster plot represent the chirp-pulses. The smaller sixth peak suggests a few chirps contained six pulses. These chirp-pulses exhibited a longer pulse interval compared to the following trill-pulses and pulses found in the A4-A5cut recording. In *T. commodus*, trills contain a variable pulse number. As the timing of the trill-pulses is measured relative to the reference-chirps, the temporal structure of trills is only obvious in the raster plot between 350 and 700 ms. The decrease in the black area of the histogram signifies the end of the reference-phrases between 1500 and 2000 ms, while the occurrence of subsequent phrases is indicated by the blue-shaded area. The transition between the phrases is indicated by a less intense dot pattern in the raster plot.

In the A4-A5cut recording, the correlograms and raster plot are based on the start of the sequences. The sequence-pulses are reflected in the peaks of the correlograms and the corresponding vertical bands in the raster plot. The interval of sequence-pulses was shorter than for chirp-pulses and there was no change of pulse form. Most sequences ended before 500 ms, as indicated by the falling light grey area in the correlogram. The timing of the subsequent sequences in the correlogram and raster plot became more scattered relative to the start of the reference-sequences, and also due to the variable pulse number in the sequences. While the song structure changed dramatically after the operation, at least in this male the interval between the last sound pulse of a trill and the start of the subsequent chirp in the control group and the interval between the last pulse of a sequence and the start of the subsequent sequence in the lesioned animal remained stable and was around 100 ms to 150 ms. This is indicated by the strict timing of the trill-pulses before the reference-chirps and the reference-pulses before the reference-sequences in the raster plot, respectively.

In 5 males, 1054 phrases (1054 chirps, 5967 chirp-pulses, 2301 trills, 26,372 trill-pulses) before the lesion and 6114 sequences with 43,706 pulses after the lesion were analysed (Fig. 7c). Comparing the temporal parameters of sequences to chirps and trills, revealed that all the pulse parameters of the sequences were in between those of chirps and trills: pulse duration (chirp: 31.6 ± 2.4 ms, trill: 24.9 ± 3.8 ms, sequence: 27.0 ± 5.1 ms, P(chirp vs trill) = 0.01, P(chirp vs sequence) = 0.102, P(trill vs sequence) = 0.493); pulse interval (chirp: 28.5 ± 3.5 ms, trill: 12.4 ± 2.3 ms, sequence: 16.4 ± 5.0 ms, P(chirp vs trill) < 0.001, P(chirp vs sequence) < 0.001, P(trill vs sequence) = 0.146); pulse period (chirp: 59.9 ± 4.0 ms, trill: 37.4 ± 4.2 ms, sequence: 43.9 ± 3.5 ms, P(chirp vs trill) < 0.001, P(chirp vs sequence) < 0.001, P(trill vs sequence) = 0.031). Also, the pulse number (chirp: 5.5 ± 1.0 pulses/chirp, trill: 11.5 ± 9.0 pulses/trill, sequence: 9.7 ± 7.5 pulses/sequence, P(chirp vs trill) = 0.176, P(chirp vs sequence) = 0.248, P(trill vs sequence) = 0.739) showed an intermediate value. In terms of song structure, sequence duration (412.0 ± 335.0 ms) was not significantly different from chirp duration (356.9 ± 34.8 ms, *p* = 0.724) or trill duration (393.8 ± 265.9 ms, *p* = 0.927). Sequence interval (229.4 ± 46.2 ms) and period (640.2 ± 302.9 ms) were close to trill interval (268.2 ± 156.3 ms, *p* = 0.609) and period (669.3 ± 409.9 ms, *p* = 0.901).

In summary, as a consequence of the A4-A5 lesion in *T. commodus*, the two typical song components, chirps and trills, were no longer generated and the calling song was composed of short sequences of sound pulses with a variable number of pulses.

## No change in calling song pattern after A5-A6 lesion in *Teleogryllus commodus*

**Fig. 8:**
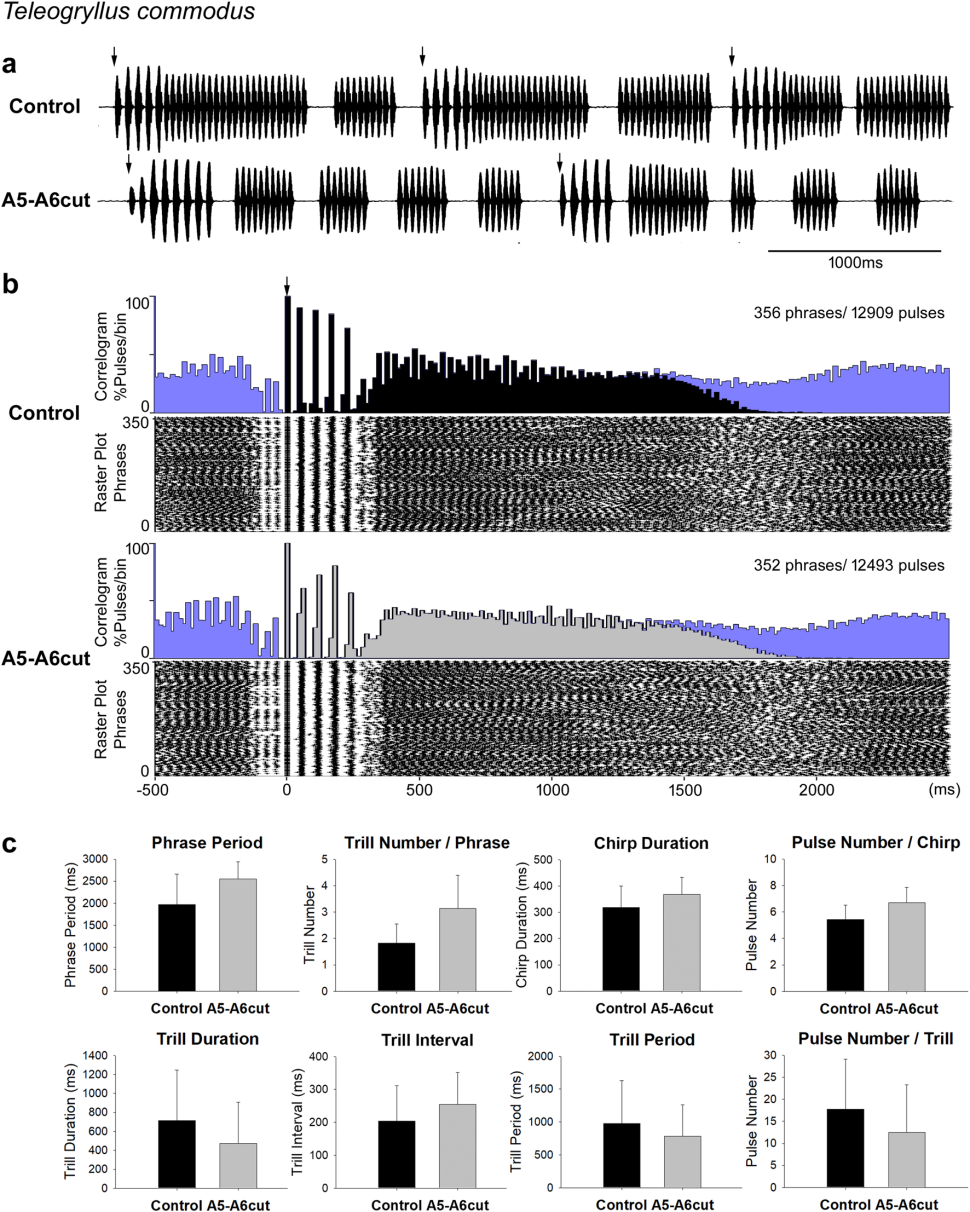
Calling song before and after the A5-A6 lesion in *T. commodus.* **a** Song before (top) and after the lesion (bottom). Arrows indicate the start of each phrase, taken as reference for the histograms. **b** Cross-correlogram and raster plot of 10-min song recordings of one male before and after the lesion over a time scale from -500 ms to 2500 ms. Black (control) and light grey shade (A5-A6cut) represent the reference-phrases in correlograms and blue shading indicates the pulses of the subsequent (to the right) or previous (to the left) phrases. Arrow indicates the start of phrases. **c** Temporal parameters of calling song before and after the lesion in 5 males. No significant differences occurred between the intact and A5-A6cut group

Males of *T. commodus* generated normal calling song phrases containing chirps and trills after the connectives between A5 and A6 were severed (A5-A6cut, *n* = 5, Fig. 8). The overall song structure was not changed after the operation (Fig. 8a). From the cross correlogram and raster plot drawn from 10-min recordings of one male, both recordings show the chirp and the trill components (Fig. 8b). At 0 ms, phrases started with chirps with a longer pulse interval. These chirps, with a stable pulse period, align in the raster plot as five bands and show as five peaks in the correlogram in both recordings. The following trill-pulses show shorter pulse intervals and only the bands of the trill-pulses after the chirps are obvious in the raster plot (between 300 and 1000 ms) because of the variable pulse number in trills. The reference-phrases ended around 1500 ms to 2000 ms, as indicated by the reduced number of pulses in raster plot and the falling light grey area in correlogram. In this male, some of the last trills in the phrases occurred with short intervals to the subsequent phrases, indicated by three light bands of dots in the raster plot between − 150 ms and 0 ms.

In 5 males, 900 phrases (900 chirps, 4810 chirp-pulses, 1526 trills, 23,577 trill-pulses) before lesion and 651 phrases (651 chirps, 4254 chirp-pulses, 2074 trills, 20,922 trill-pulses) after lesion were analysed statistically (Fig. 8c). The calling song structure of A5-A6cut males showed no significant difference in phrase period (control: 1968.5 ± 688.8 ms, A5-A6cut: 2548.2 ± 389.7 ms, *p* = 0.140), chirp duration (control: 318.5 ± 81.7 ms, A5-A6cut: 367.9 ± 65.4 ms, *p* = 0.322) and trill number in phrases (control: 1.8 ± 0.7 trills/phrase, A5-A6cut: 3.1 ± 1.3 trills/phrase, *p* = 0.076). The trill parameters like trill duration (control: 713.5 ± 533.4 ms, A5-A6cut: 474.6 ± 435.2 ms, *p* = 0.460), trill interval (control: 204.6 ± 107.0 ms, A5-A6cut: 255.0 ± 96.0 ms, *p* = 0.456), trill period (control: 979.9 ± 652.3 ms, A5-A6cut: 781.6 ± 481.3 ms, *p* = 0.599) showed no significant difference after A5-A6 lesion. Finally, the pulse number in chirps (control: 5.4 ± 1.1 pulses/chirp, A5-A6cut: 6.7 ± 1.1 pulses/chirp, *p* = 0.111) and pulse number in trills (control: 17.8 ± 11.3 pulses/trill, A5-A6cut: 12.5 ± 10.8 pulses/trill, *p* = 0.469) were not significantly changed after the lesion.

Also, the pulse parameters of chirps and trills produced by males with an A5-A6 lesion were not significantly different from intact males. For chirps, chirp-pulse duration (control: 31.3 ± 4.6 ms, A5-A6cut: 27.3 ± 5.5 ms, *p* = 0.244), chirp-pulse interval (control: 32.7 ± 4.9 ms, A5-A6cut: 31.7 ± 5.3 ms, *p* = 0.767), and chirp-pulse period (control: 63.9 ± 6.6 ms, A5-A6cut: 58.9 ± 4.7 ms, *p* = 0.206) did not change between the two groups. Pulse parameters in trills also showed no significant difference before and after the A5-A6 lesion: trill pulse duration (control: 25.9 ± 4.1 ms, A5-A6cut: 22.1 ± 4.2 ms, *p* = 0.195), trill pulse interval (control: 13.6 ± 3.1 ms, A5-A6cut: 16.5 ± 4.7 ms, *p* = 0.267), and trill pulse period (control: 39.5 ± 3.7 ms, A5-A6cut: 38.8 ± 2.9 ms, *p* = 0.753).

To summarise, the calling song of *T. commodus* was not altered by the lesion between A5 and A6 in terms of phrase structure and the temporal pulse parameters.

## Discussion

Recent lesion studies (Schöneich and Hedwig 2011; Jacob and Hedwig 2016) revealed that the singing-CPG in the field cricket *G. bimaculatus* is organized along the abdominal nerve cord and not located in the thoracic ganglia as previously suggested (Huber 1960, 1963; Hennig and Otto 1996). To investigate if the neural network organization for song pattern generation is similar and conserved among cricket species, we performed lesion experiments on four species from different clades (Gray et al. 2020) generating very different species-specific calling songs with pulses organized in chirps, trills or phrases.

All four species showed the same effect on singing after a lesion of the T3-A3 connectives. Males retained the ability to raise the forewings into singing position but for the rest of their lives they failed to generate rhythmic wing movements like for singing and no longer produced any proper sound pulses. This outcome is in line with the results of acute T3-A3 connective severing experiments in *G. bimaculatus* males singing due to brain stimulation (Schöneich and Hedwig 2011) and with long-term lesion experiments (Jacob and Hedwig 2016). It provides further strong evidence that the abdominal ganglion chain is crucial for the generation of calling song in different cricket species. The results provide a deeper insight of experiments in *G. campestris* (Kutsch and Otto 1972), demonstrating that the CNS fails to generate the calling song pattern when the cervical connectives *and* the abdominal connectives are cut. Moreover, the results indicate that the control of the elevated wing position used for singing does not depend on the abdominal ganglia but rather is under control of the cephalic and/or thoracic nervous system.

After lesions to the A3-A4 connectives overall singing activity was greatly reduced. Males could still produce single sound pulses, however in all species the typical calling song structure was lost. This suggests the abdominal ganglion A3 houses neural circuits that are sufficient to control the opening-closing wing movements for pulse generation. This is in line with the results in *G. bimaculatus* (Jacob and Hedwig 2016), suggesting that the A3 ganglion is part of a pulse-timer network. This is also supported by the discovery of an ascending opener interneuron in A3 (Schöneich and Hedwig 2011, 2012), which has been identified in five cricket species (Jacob and Hedwig 2019, 2020) and is an element of the singing-CPG. It shows bursts of spikes preceding wing opener motoneuron activity and elicits singing motor activity upon current injection.

A lesion to the A4-A5 connectives altered the song in *G. assimilis*, *T. oceanicus*, and *T. commodus.* In *G*. *bimaculatus*, such a lesion led to a breakdown of the calling song chirp structure with chirp duration considerably extended and coupled to a more variable chirp period (Jacob and Hedwig 2016). After the lesion also, the chirps in *G. assimilis* exhibited an increased pulse number and a longer chirp duration, while the rather long variable chirp period was not altered.

The effect of the A4-A5 lesion on the calling songs of the phrase-producing *Teleogryllus* species was dramatic. The organised chirp and trill components of the phrases disappeared and instead males generated repetitions of short sequences of sound pulses with a variable pulse number. These newly generated pulse sequences showed almost all pulse parameters in between the normal chirp-pulses and trill-pulses. The comparison of pulse number in chirps, trills, and sequences before and after the lesion may reveal the effect of the lesion on the song structure in more detail. In normal calling song, both species produced 4–6 pulses in chirps, however, the number of pulses/trill in *T. commodus* (11.5 ± 9.0) was higher than in *T. oceanicus* (2.3 ± 0.5). After the A4-A5 lesion, the number of pulses/sequences was in between the normal number of pulses/chirp and pulses/trill, still the number of pulses/sequence was higher in *T. commodus* (9.7 ± 7.5) than in *T. oceanicus* (3.0 ± 0.6), and more similar to the normal trill situation in each species. This may indicate that a property of the network that determines the pulse number per trill as a species-specific feature, was partially retained after the A4-A5 lesion.

Overall, the A4-A5 lesions demonstrated that the remaining network can no longer generate the coordinated pattern of chirps and trills, and that at least the A5 ganglion contributes to the generation of the phrases, housing a neural mechanism that controls the generation of chirps and trills. This either could be due to chirps and trills being driven by different excitation levels of one neuron or network or it could be due to alternating activity levels of two functionally separate networks. It also demonstrates that ganglia A3 and A4 together can generate sequences of sound pulses, according to the implications from the *G. bimaculatus* experiments that the pulse-timer network extends over the A3 and A4 ganglion (Jacob and Hedwig 2016).

Interestingly in *G. rubens*, no significant change in the trill structure of the song or of pulse parameters occurred. Even in intact males, the trill pattern was variable and not precisely structured. In the evolution of cricket calling songs, a train of pulses may represent the original ancestral condition from which more complex songs with 2–3 rhythms evolved (Otte 1992). The complex patterns indicate a coupling of different timer networks to generate the more complex motor output (Jacob and Hedwig 2016). Evolutionary analysis of cricket taxa puts *G. rubens* in a rather original line (Gray et al. 2020) and here a network in A5 contributing to the timing of trills like to the timing of chirps and phrases in the other species may actually not be present.

In all 4 species, lesions to the A5–A6 connectives had no or minor effects on the structure of the calling song. This implicates that the A6 and the TAG are not required for calling song pattern generation and that sensory information on the presence of a spermatophore is not required, which is different to previous conclusions (Huber 1960, 1963). It also implicates that the effect of the A4-A5 lesion can be attributed to a contribution of the A5 ganglion to pattern generation, corresponding to the organization of the chirp-timer network along abdominal ganglia in *G. bimaculatus* (Jacob and Hedwig 2016). The lesions applied here were incisions of the connectives between ganglia. To interpret the role of each abdominal ganglion in more detail, hemisecting the ganglia in these four species as in *G. bimaculatus* (Jacob and Hedwig 2016), might provide further information on the organization of the calling song network.

The similar effects of lesions to the abdominal ganglion chain indicate that the overall organization of the singing-CPG is conserved among cricket species, besides the species-specific song structure. The pattern generating network underlying singing stretches over the A3, A4 and A5 ganglia. The temporal structure of the singing activity is getting more complex with more posterior ganglia contributing to motor pattern generation, indicating that the recruitment of network components in the more posterior abdominal ganglia may have supported the evolution of more complex song structures. It may appear surprising that the cricket singing-CPG extends over the abdominal ganglia and is not located in the second thoracic ganglion, which houses the motoneurons innervating the singing muscles. It was proposed singing might be closely linked to the flight motor pattern (Huber 1962), however functionally separated interneuron populations (Hennig (1990) and the abdominal organization of the singing network make this unlikely. In the ventilatory system of locusts, each abdominal ganglion has the ability to autonomously generate the ventilatory motor cycle (Lewis et al. 1973). One possibility that receives support from behavioural studies and EMG recordings (Kutsch 1969b, Paripovic et al. 1996), and recent electrophysiological experiments (Schöneich and Hedwig 2019) is that in crickets elements of the ventilatory pattern generator network in the abdominal ganglion chain supported the evolution of the singing network. Besides this, the abdominal ganglia in other insects and invertebrates also show autonomous generation of motor activity, which during locomotor activity is coordinated along the chain of abdominal ganglia. In larval Drosophila waves of fictive motor activity, akin to control forward crawling movements, start in the most posterior segments and spread anteriorly (Pulver et al. 2015). Also in the swimmeret system of crayfish intersegmentally coordinated motor activity starts from the posterior fifth abdominal ganglion and spreads anteriorly (Ikeda and Wiersma 1964) and is based on the coordinated activation of modular organized pattern generator networks in the abdominal ganglia A2 to A5 (Mulloney and Smarandache-Wellmann 2012). Thus, a common ancient abdominal network organization may have provided a neuronal substrate for the evolution of the singing motor patterns in crickets. From an evolutionary perspective, it may be interesting to apply similar lesion studies to the trigonidiine Laupala crickets which, due to their rapid speciation based on changes in pulse rate, are in the focus of genetic studies (Shaw and Danley 2003); or to acoustically communicating bush-crickets, which also use their wings for sound production and share common ancestors with crickets but may have evolved acoustic communication independently (Desutter-Grandcolas 2003).

Lesion experiments in *G. campestris* once excluded the involvement of abdominal ganglia in singing behaviour; however, in those experiments, the exact sites of lesions to the abdominal connectives were not specified (Huber 1960, 1963). As such lesions show very different results depending on the connectives severed and, e.g. lesions posterior to the A5 ganglion have no effect on singing, the interpretation of these early experiments may have been confounded. It still might be possible that the singing-CPG in *G. campestris* is organized in a different way, however considering the results by Kutsch and Otto (1972), and the effect of the lesions in the four species studied and in *G. bimaculatus* (Jacob and Hedwig 2016), and the close phylogenetic relationship between *G. bimaculatus* and *G. campestris* (Huang et al. 2000; Desutter-Grandcolas and Robillard 2003), a different organization of the singing network in *G. campestris* seems very unlikely.

Lesions applied to the insect central nervous system have been an important approach to study behaviour and the gross organization of the underlying neural networks. In the study of grasshopper stridulation, it narrowed down the ganglia housing the singing CPG to the 2nd and 3rd thoracic ganglion (Hedwig 1986), while the hemi-ganglionic organization of the singing-CPG in the 3rd thoracic ganglion was revealed (Ronacher (1989, 1991; Fries and Elsner 1996; Heinrich and Elsner 1997). A hemiganglionic organisation of the pattern generator in thoracic ganglia was also demonstrated for the locust flight system by means of severing pairs of connectives and hemisection of thoracic ganglia (Wolf et al. 1988; Ronacher et al. 1988). Although lesion experiments may be regarded as an outdated approach, they still provide valuable information on the organization of motor pattern generating networks (Hückesfeld et al. 2015). The current and previous examples demonstrate that they can substantially contribute to our understanding of nervous systems.

## Data Availability

Details for data can be obtained from the authors.

## Abbreviations

A3 … A6: Third … sixth abdominal ganglion
A3-A4cut: Lesion between 3rd and 4th abdominal ganglion
Singing-CPG: Central Pattern Generator for singing
TAG: Terminal abdominal ganglion
T3: Metathoracic ganglion
